# Prognostic Implication of Longitudinal Changes of Left Ventricular Global Strain After Chemotherapy in Cardiac Light Chain Amyloidosis

**DOI:** 10.3389/fcvm.2022.904878

**Published:** 2022-06-24

**Authors:** Minjung Bak, Darae Kim, Jin-Oh Choi, Kihyun Kim, Seok Jin Kim, Eun-Seok Jeon

**Affiliations:** ^1^Division of Cardiology, Department of Medicine, Samsung Medical Center, Heart Vascular Stroke Institute, Sungkyunkwan University School of Medicine, Seoul, South Korea; ^2^Divsion of Hematology and Oncology, Department of Medicine, Samsung Medical Center, Sungkyunkwan University School of Medicine, Seoul, South Korea

**Keywords:** cardiac amyloidosis, global longitudinal strain, cardiac organ response, mortality, chemotherapy

## Abstract

**Aim:**

Cardiac involvement is the main prognostic determinant in AL amyloidosis. We sought to determine the prognostic significance of longitudinal change of left ventricular (LV) global longitudinal strain (GLS) in cardiac light chain (AL) amyloidosis patients undergoing chemotherapy.

**Methods and Result:**

We retrospectively investigated 117 cardiac AL amyloidosis patients who underwent chemotherapy from 2005 to 2019. All patients underwent comprehensive 2D conventional transthoracic echocardiography at baseline and after completion of first-line chemotherapy. Speckle tracking analysis of images was performed offline. Absolute value of LV GLS was expressed as [LV GLS] and change of [LV GLS] after chemotherapy was expressed as Δ [LV GLS]. Clinical outcomes including cardiac response and all-cause mortality were analyzed.

Baseline clinical and echocardiographic parameters were similar in patients with and without CR. Δ [LV GLS] significantly differed between the CR and non-CR groups (0.4 ± 2.8% in the CR group vs. −0.6 ± 2.5% in the non-CR group, *P*-value = 0.046). Δ [LV GLS] showed satisfactory predictive performance for all-cause mortality (cut-off value = 0.8%, AUC 0.643, 95% CI [0.537–0.748]). Adding Δ [LV GLS] to the Mayo stage + pre-chemotherapy [LV GLS] model showed incremental prognostic value (C-index: 0.637 vs. 0.708; Relative Integrated Discrimination Index 0.07, *P*-value = 0.003; Net Reclassification Improvement 0.54, *P-*value < 0.001). Δ [LV GLS] showed good correlation with cardiac response (AUC 0.820, 95% CI [0.737–0.904]).

**Conclusion:**

In cardiac amyloidosis patients who underwent chemotherapy, longitudinal change of [LV GLS] after chemotherapy showed significant association with overall survival as well as cardiac response.

## Introduction

Due to recent improvements in chemotherapy, the prognosis of patients with cardiac involvement has improved (1–4). Cardiac involvement is the main driver of prognoses in patients with light chain (AL) amyloidosis, and staging for cardiac involvement is a critical part of risk stratification. Current guidelines include cardiac biomarkers, N-terminal prohormone of brain natriuretic peptide (NT pro-BNP), and troponin T for risk stratification, which is critical for decision-making regarding aggressive strategies or modifications to the chemotherapeutic approach (5, 6). Before development of therapeutic agents, therapeutic trials including advanced cardiac involvement were limited (7, 8), and patients with cardiac AL amyloidosis had poor survival outcomes. In recent years, with the advent of immune-modulatory drugs and proteasome inhibitors, patients contraindicated to stem cell transplantation could be treated, and improvement of treatment outcomes for cardiac AL amyloidosis has been observed (9–11). Recent studies reported a prognostic incremental value of left ventricular (LV) global longitudinal strain (GLS) in cardiac AL amyloidosis patients (12–17).

The aim of cytotoxic therapy in AL amyloidosis patients is to reduce or halt further production of the toxic light chains. Prolonged survival and recovery of involved organs are expected with cessation of further production of toxic light chains. Cardiac response imposes substantial prognostic implications on mortality outcomes (18). In this study, we sought to investigate if longitudinal changes of LV GLS after the first line of chemotherapy predicted cardiac response and overall survival in patients with cardiac AL amyloidosis.

## Materials and Methods

### Patient Selection

Consecutive patients with biopsy-proven AL amyloidosis from 1995 to 2019 at Samsung Medical Center who were registered in a prospective amyloid registry were analyzed. The diagnosis of AL amyloidosis required tissue confirmation of amyloid deposits or fibrils by apple-green birefringence with Congo red staining, kappa or lambda restriction by immunohistochemistry in at least one involved organ, and evidence of monoclonal gammopathy (19). A total of 344 patients was diagnosed with cardiac AL amyloidosis. Cardiac involvement was defined by the presence of amyloid deposits on endomyocardial biopsy or based on imaging findings in affirmed amyloidosis patients as previously described (17). Among the 344 patients, 301 (88%) underwent chemotherapy. All patients underwent echocardiography at the time of the diagnosis. Patients who did not survive until the response date, patients without follow-up echocardiography after the first cycle of chemotherapy, or patients with poor echocardiography image quality were excluded. Finally, this study included a total of 117 cardiac AL amyloidosis patients. Among these 117 patients, 83 and 46 were confirmed by endomyocardial biopsy and image findings, respectively. Baseline clinical characteristics and laboratory, imaging, and mortality data were collected prospectively from the registry at the initiation of first-line chemotherapy.

### Outcomes and Follow-Up

All patients were regularly followed up after diagnosis. Hematologic responses were determined by the change in the difference between involved and uninvolved free light chains (dFLC) according to consensus guidelines (20) at 3 and 6 months after the initiation of first-line chemotherapy. A complete hematologic response (CR) was defined as the absence of monoclonal protein in serum and urine by immunofixation electrophoresis and a normal serum-free light chain (FLC) ratio (20, 21). Very good partial response (VGPR), partial response (PR), and no response (NR) were defined as a reduction of dFLC < 4 mg/dL, ≥ 50% reduction in dFLC, and < 50% reduction in dFLC, respectively. Any patients who did not achieve CR after the first line of chemotherapy were classified into the non-CR group. Baseline clinical and echocardiographic characteristics were compared between the CR and non-CR groups. Laboratory data and echocardiographic parameters related to treatment response were compared between pre-chemotherapy and post-chemotherapy after stratifying according to CR.

Cardiac response was defined as a reduction of NT-proBNP > 30% of baseline level, as described in the recent consensus criteria, at 12 months after initiation of the first line of chemotherapy (18). The NT pro-BNP level at initiation of first-line chemotherapy was used as the initial baseline measurement.

Prospective follow-up began on the diagnosis date. All-cause death was the primary outcome. The mortality endpoint was defined as the time to all-cause death from follow-up echocardiography for all deceased patients and the time to censoring date (October 2020) for all other patients. Occurrence and date of death and ongoing survival status were routinely monitored by regular visits or telephone calls. The median follow up duration from initial diagnosis to censor day was 24.8 months (quartile range, 9.1–45.9 months) and median follow-up duration from follow up echocardiography to censor day was 15.0 months (quartile range, 1.8–34.7 months).

### Echocardiography

All patients underwent comprehensive two-dimensional conventional transthoracic echocardiography (TTE) both at the initiation and after completion of the first line of chemotherapy (median time interval: 7.0 months) according to the previous guideline (22). Left ventricular (LV) end-diastolic and end-systolic diameter, LV septal wall thickness, and posterior wall thickness were measured from the parasternal long-axis view. LV ejection fraction was calculated from 2-dimensional recordings using the modified biplane Simpson’s method. Left atrial (LA) volume was assessed by the modified biplane area-length method. The ratio of early transmitral flow to early septal mitral annular diastolic velocity (E/e’) was measured as an index of LV filling pressure. Right ventricular systolic pressure (RVSP) was estimated by tricuspid regurgitation jet maximal velocity.

For two-dimensional strain analysis, images were analyzed offline using vendor-independent 2D Cardiac Performance Analysis software, version 1.1.3 (Tom Tec Imaging Systems, Munich, Germany). The endocardial border of the LV was manually traced from three apical views (apical 2-, 3-, and 4-chamber views) and averaged to obtain LV GLS. Absolute value of LV GLS was expressed as [LV GLS], and change in [LV GLS] after chemotherapy was expressed as Δ [LV GLS] (post-chemotherapy [LV GLS]—pre-chemotherapy [LV GLS]). Basal segmental value was the average of 6 segments in the LV basal area, and apical segmental value was the average of 4 segments in the LV apical lesion. LV GLS was measured by two well-trained operators (MB, DK). All echocardiographic measurements were conducted with the operator blinded to the clinical and outcome data. Inter-observer and intra-observer variability were assessed using the intra-class correlation coefficient (ICC). The inter-observer variability was calculated by comparing GLS calculations in 15 randomly selected patients. Intra-observer variability was calculated by repeated measurements in 15 patients at 4 weeks after the initial measurement.

### Statistical Analysis

For continuous variable comparison between the CR and non-CR groups, Student’s *t*-test or Mann-Whitney U test was used to determine differences and presented by mean ± standard deviation (SD) or median (interquartile range), respectively. Differences in categorical variables between the CR and non-CR groups were analyzed by Chi-square test or Fisher’s exact test and presented by numbers and relative frequencies (%). The Shapiro-Wilk test was used for normality test of continuous variables. Paired *t*-test and Wilcoxon test were used for paired continuous variable comparison between pre-chemotherapy and post-chemotherapy.

The optimal cutoff values of Δ [LV GLS] for predicting all-cause death were calculated to maximize the product of sensitivity and specificity using receiver operating characteristic (ROC) curves. The cumulative incidence of all deaths was presented as the Kaplan-Meier estimate and compared using a log-rank test. Cox proportional hazards regression was used to calculate hazard ratios (HRs) and 95% confidence intervals (CIs) to compare the risk of all deaths between groups. The time interval for survival analysis was applied with the time interval between follow-up echocardiography and censor date. The C-statistic was used to evaluate the predictive and explanatory power of Δ [LV GLS], and the Delong method evaluated the accuracy of the C-statistic. The additive prognostic implications of Δ [LV GLS] and pre-chemotherapy [LV GLS] were evaluated by assessing improvements in the discriminant and reclassification ability of the models compared with the Mayo 2012 stage model using the category-free Net Reclassification Index and integrated discrimination improvement. In variable selection for multivariable logistic regression, the criterion for entry was *P*-value < 0.05, and the criterion for elimination was *P*-value > 0.10. Pearson’s product moment correlation coefficient was used in correlation analysis. All probability values were two-sided, and *P*-values < 0.05 were considered statistically significant. Statistical analyses were performed using R Statistical Software (version 3.6.0; R Foundation for Statistical Computing, Vienna, Austria) and SPSS Statistics 26 (SPSS Inc., Chicago, IL).

### Ethical Consideration

The study protocol was approved and the requirement for informed consent of the individual patients was waived by the Institutional Review Board of Samsung Medical Center. This study was conducted according to the principles of the Declaration of Helsinki.

## Results

### Baseline Characteristics and Clinical Outcome

The mean age of patients was 60.3 years, and 54.7% were male. In our cohort, 53 patients (46.9%) were classified as Mayo stage IV and 46 patients (40.7%) as Mayo stage III at baseline. A total of 72 patients (62.6%) had dyspnea greater than NYHA class II at the time of diagnosis. The baseline [LV GLS] was 12.0%, and mean Δ [LV GLS] was −0.3% in the total study population. Regional LV GLS showed an apical sparing pattern with lowest value basal [LV GLS]. During the median follow-up of 25 months, a total of 42 patients died. Five-year all-cause mortality was 52% (*n* = 39). Seventy-four patients (64.9%) achieved cardiac organ response between the initiation of chemotherapy and determination of cardiac response, and 34 patients (29.1%) achieved complete hematologic response between the initiation of chemotherapy and determination of hematologic response.

### Baseline Characteristics Comparison Between the CR and Non-CR Groups and Post-chemotherapy Change

There were no statistical differences between the CR group and the non-CR group in baseline clinical characteristics (Table 1). After chemotherapy, kappa free light chain and NT-proBNP level significantly decreased compared to baseline levels in the CR group compared to the non-CR group. Lambda free light chain and dFLC level significantly decreased in both CR and non-CR group. In the non-CR group, septal wall thickness was increased and LV ejection fraction was decreased after chemotherapy compared to baseline (Table 2).

**TABLE 1:** Comparison of baseline characteristics between CR and non-CR groups.

	CR group (*n* = 34)	Non-CR group (*n* = 83)	*P*-value	Total (*n* = 117)
Age	59.9 ± 9.4	60.5 ± 10.2	0.757	60.3 ± 9.9
Men, *n* (%)	17 (50.0%)	47 (56.6%)	0.653	64 (54.7%)
NYHA classification			0.648	
II	17 (50.0%)	31 (38.3%)		48 (41.7%)
III	6 (17.6%)	12 (14.8%)		18 (15.7%)
IV	1 (2.9%)	5 (6.2%)		6 (5.2%)
Involved FLC, *n* (%)			0.690	
Lambda	27 (81.8%)	61 (76.2%)		88 (77.9%)
Kappa	6 (18.2%)	19 (23.8%)		25 (22.1%)
Initial dFLC, mg/dL	225.1 [145.1–570.7]	346.8 [142.9–847.1]	0.290	306.6 [143.2–734.5]
Revised mayo stage, *n* (%)			0.505	
II	4 (12.1%)	8 (10.0%)		12 (10.6%)
III	10 (30.3%)	36 (45.0%)		46 (40.7%)
IV	18 (54.5%)	35 (43.8%)		53 (46.9%)
Other organ involvement, *n* (%)				
Kidney	15 (44.1%)	36 (43.4%)	1.000	51 (43.6%)
Liver	4 (11.8%)	6 (7.2%)	0.665	10 (8.5%)
NT-proBNP, pg/dL	4756.0 [1682.0–7800.0]	2844.0 [1445.5–6474.0]	0.168	2997.0 [1577.0–6891.0]
Troponin T, ng/dL	0.077 [0.038–0.114]	0.079 [0.042–0.119]	0.785	0.078 [0.041–0.117]
Creatinine (mg/dL)	0.9 [0.7–1.1]	1.0 [0.8–1.2]	0.446	0.9 [0.7–1.2]
eGFR (mL/min/1.73m^2^)	74.8 [63.0–85.8]	75.9 [57.8–92.6]	0.879	75.2 [59.1–92.2]
Atrial fibrillation	5 (14.7%)	14 (16.9%)	0.991	19 (16.2%)
Hypertension	6 (17.6%)	16 (19.3%)	1.000	22 (18.8%)
Diabetes mellitus	1 (2.9%)	9 (10.8%)	0.306	10 (8.5%)
*Chemotherapy*				
Bortezomib	24 (70.6%)	41 (49.4%)	0.059	65 (55.6%)
Alkylating agent	19 (55.9%)	42 (50.6%)	0.753	61 (52.1%)
Stem cell transplantation	8 (23.5%)	21 (25.3%)	1.000	29 (24.8%)
*Chemo-combination*				
Bortezomib + melphalan + steroid	12 (35.3%)	20 (24.1%)		32 (27.4%)
Bortezomib + dexa	9 (26.5%)	10 (12.0%)		19 (16.2%)
Melphalan + dexa	6 (17.6%)	10 (12.0%)		16 (13.7%)
Thalidomide + dexa	1 (2.9%)	14 (16.9%)		15 (12.8%)
CyBorD	3 (8.8%)	10 (12.0%)		13 (11.1%)
Cytoxan + steroid	1 (2.9%)	12 (14.5%)		13 (11.1%)
Etc.	2 (5.9%)	7 (8.4%)		9 (7.7%)

*CyBorD, cyclophosphamide-bortezomib-dexamethasone; CR, complete hematologic response; dFLC, difference between involved and uninvolved serum free light chains; eGFR, estimated glomerular filtration rate; FLC, free light chain; NT-proBNP, N-terminal pro-brain natriuretic peptide; NYHA, New York Heart Association.*

**TABLE 2:** Pre- and post-chemotherapy (CTx) characteristics stratified by hematologic response.

	CR (*n* = 34)		Non-CR (*n* = 83)	
	Pre-CTx	Post-CTx	Pre-CTx	Post-CTx
Maximum FLC level (mg/L), K	21.6 [13.0–35.8]	16.7 [10.5–21.1]^§§^	17.1 [11.2–55.8]	20.4 [12.0–44.0]
Maximum FLC level (mg/L), L	225.2 [74.2–623.0]	17.4 [13.3–24.9]^§§§^	195.9 [45.8–577.8]	53.1 [24.8–223.7]^§§§^ ***
dFLC (mg/L)	437.1 ± 481.8	14.2 ± 35.6^§§§^	882.9 ± 1510.9*	341.1 ± 819.3^§§§^ **
Troponin T, ng/dL	0.059 [0.038–0.113]	0.050 [0.033–0.075]	0.080 [0.051–0.123]	0.059 [0.045–0.116]
NT-proBNP, pg/dL	4756.0 [1682.0–7800.0]	2764.0 [1445.5–6474.0]^§§§^	1167.0 [488.0–2877.0]	1616.0 [656.5–6378.0]
LV EDD, mm	45.8 ± 4.2	45.8 ± 5.0	45.7 ± 4.7	45.0 ± 4.8
LV ESD, mm	30.0 [28.0–33.0]	31.1 [29.0–33.5]	29.0 [26.0–32.0]	29.8 [27.0–32.0]
Septal wall thickness, mm	12.0 [12.0–14.0]	12.8 [12.0–13.2]	12.0 [11.0–13.0]	12.5 [11.9–14.2]^§§^
Posterior wall thickness, mm	12.0 [11.0–13.0]	12.8 [12.0–13.4]	12.0 [11.0–13.0]	12.0 [11.8–14.0]
LV ejection fraction,%	56.0 [54.0–61.0]	54.4 [45.8–61.0]	61.0 [54.0–64.5]	57.0 [49.8–64.0]^§§^
LA volume index, ml/m^2^	52.7 [44.0–62.0]	49.8 [43.4–58.0]	44.9 [37.8–56.3]	45.7 [39.0–52.9]
Septal e’, m/s	0.039 ± 0.013	0.040 ± 0.012	0.045 ± 0.012*	0.042 ± 0.013
E/e’	22.0 [16.1–26.2]	19.5 [14.5–22.8]^§^	17.9 [12.6–24.7]	19.5 [13.1–23.5]
RVSP, mmHg	38.0 [28.5–44.0]	30.5 [27.0–39.5]^§^	33.0 [27.0–42.5]	33.0 [29.0–42.0]
[*LVGLS*],%	11.5 [9.7–13.4]	11.0 [9.7–13.4]	12.6 [9.7–14.5]	11.6 [8.8–14.0]^§^
[*LVGLS*] basal.%	6.0 ± 4.0	6.4 ± 4.2	6.4 ± 3.4	6.2 ± 3.7
[*LVGLS*] apical,%	18.6 ± 5.6	18.3 ± 5.7	19.5 ± 5.5	18.5 ± 5.5^§^
RRSR	3.4 [2.5–5.0]	2.8 [2.3–4.1]	3.1 [2.2–4.6]	2.8 [2.1–4.2]

*dFLC, difference between involved and uninvolved free light chains; E, early diastolic mitral inflow velocity; e’, early diastolic mitral annular tissue velocity; EDD, end diastolic dimension; ESD, end systolic dimension; FLC, free light chain; K, kappa; L, lambda; LA, left atrium; LV, left ventricular; [LV GLS], absolute value of left ventricular global longitudinal strain; NT-proBNP, N-terminal pro-brain natriuretic peptide; RRSR, relative regional strain ratio (average apical LS/average basal LS); RVSP, right ventricular systolic pressure.*

*P < 0.05 compared to CR group, ^§^P < 0.05 compared to pre-CTx.

**P < 0.005 compared to CR group, ^§§^P < 0.005 compared to pre-CTx.

***P < 0.001 compared to CR group, ^§§§^P < 0.001 compared to pre-CTx.

**FIGURE 1:**
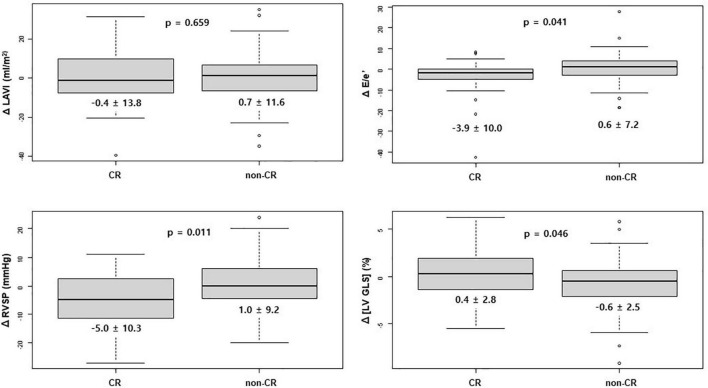
Change in echocardiographic value after chemotherapy between the CR and non-CR groups. CR, complete response; E, early diastolic mitral inflow velocity; e’, early diastolic mitral annular tissue velocity; LAVI, left atrium volume index; [LV GLS], absolute value of left ventricular global longitudinal strain; RVSP, right ventricular systolic pressure.

In the CR group, E/e’ and RVSP significantly improved between initial echocardiography and follow-up echocardiography, while such changes were not observed in the non-CR group. [LV GLS] after chemotherapy significantly deteriorated in the non-CR group, while there was no significant change in the CR group (Figure 1 and Table 2).

### Prognostic Implications of Δ [LV GLS] in Overall Survival

**FIGURE 2:**
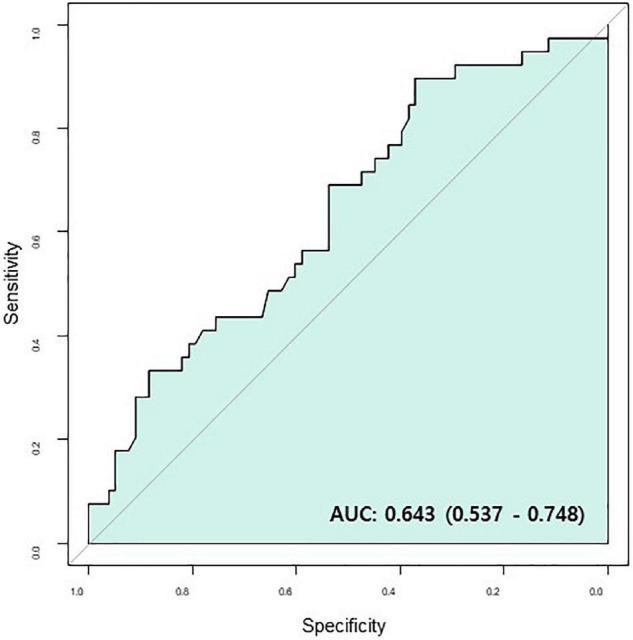
Survival ROC of Δ [LV GLS]. AUC, area under curve; CR, complete response; Δ [LV GLS], post-chemotherapy [LV GLS]—pre-chemotherapy [LV GLS]; ROC, receiver operating characteristic.

**FIGURE 3:**
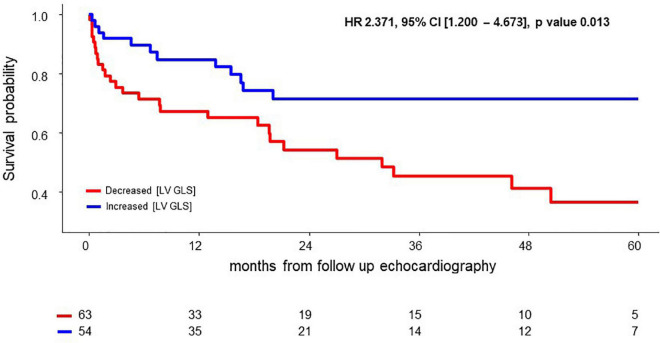
Survival difference according to Δ [LV GLS] after chemotherapy. CI, confidence interval; HR, hazard ratio; Δ [LV GLS], post-chemotherapy [LV GLS]—pre-chemotherapy [LV GLS].

In multivariable Cox regression analysis for all-cause death, change of NT-pro BNP level after chemotherapy (Post NT-proBNP/Pre NT-proBNP, adjusted HR 1.252, 95% CI [1.065–1.472], *P*-value = 0.007) and Δ [LV GLS] (adjusted HR 0.819, 95% CI [0.714–0.940], *P*-value = 0.004) were significantly associated with mortality after adjusting for relevant factors (Table 3). In survival ROC analysis, Δ [LV GLS] showed modest predictive power for mortality, with a C-index value of 0.643 (95% CI: 0.537–0.748) (Figure 2). Patients with decreased [LV GLS] after chemotherapy had worse prognosis with HR 2.371 (95% CI: 1.200–4.673, *P*-value = 0.013) compared with increased [LV GLS] group (Figure 3). The cut-off value of Δ [LV GLS] for mortality in ROC analysis was 0.8%, and patients with a value above this cut-off had a better prognosis.

**TABLE 3:** Univariable and multivariable Cox analysis of 5-year all-cause death risk according to clinical characteristics, biomarkers and echocardiographic parameters.

Variable	Univariable analysis			Multivariable analysis		
	HR	95% CI	*P*-value	HR	95% CI	*P-value*
Age, years	1.033	0.999–1.069	0.055	1.021	0.980–1.063	0.325
Sex (Men)	1.596	0.837–3.043	0.156			
Kidney involvement	0.806	0.422–1.537	0.512			
Septal wall thickness, mm	**1.184**	**1.036**–**1.353**	**0.013**	1.154	0.983–1.356	0.080
LV ejection fraction,%	0.972	0.943–1.003	0.072	**0.953**	**0.913**–**0.995**	**0.028**
E/e’	1.012	0.987–1.038	0.365	0.999	0.965–1.034	0.950
Δ troponin T, ng/dL	0.039	<0.001–7.454	0.226			
Δ dFLC, mg/L	1.000	1.000–1.000	0.852			
Post NT-proBNP/Pre NT-proBNP	**1.289**	**1.171**–**1.417**	**<0.001**	**1.252**	**1.065**–**1.472**	**0.007**
Δ [LV GLS],%	**0.844**	**0.757**–**0.940**	**0.002**	**0.819**	**0.714**–**0.940**	**0.004**

*CI, confidence interval; dFLC, difference between involved and uninvolved serum free light chains; Δ dFLC, post-chemotherapy dFLC—pre-chemotherapy dFLC; E, early diastolic mitral inflow velocity; e’, early diastolic mitral annular tissue velocity; HR, hazard ratio; LV, left ventricular; [LV GLS], absolute value of left ventricular global longitudinal strain; Δ [LV GLS], post-chemotherapy [LV GLS]—pre-chemotherapy [LV GLS]; NT-proBNP, N-terminal pro-brain natriuretic peptide; Δ troponin T, post-chemotherapy troponin T—pre-chemotherapy troponin T. The values in bold indicate statistical significance (p < 0.05).*

**FIGURE 4:**
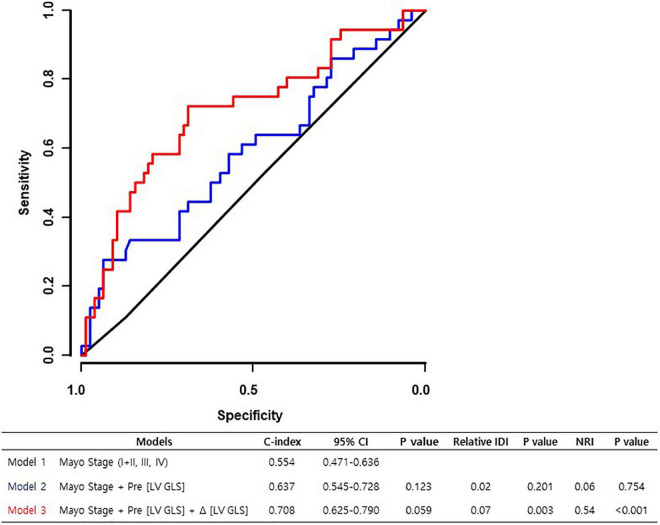
Addictive prognostic value of Δ [LV GLS] and Pre [LV GLS] in 2012 Mayo stage. C-index, harrell’s concordance index; CI, confidence interval; IDI, Integrated Discrimination Index; Δ[LV GLS], Post-chemotherapy [LV GLS]—Pre-chemotherapy [LV GLS]; NRI, Net Reclassification Improvement; Pre [LV GLS], Pre-chemotherapy [LV GLS].

Adding Δ [LV GLS] to the Mayo 2012 stage + pre-chemotherapy [LV GLS] model resulted in a distinctively better prediction model (relative IDI 0.07 and *P*-value = 0.003; NRI 0.54 and *P*-value < 0.001). On the other hand, adding pre-chemotherapy [LV GLS] to the Mayo 2012 stage model did not yield a better prognostic value (relative IDI 0.02 and *P*-value = 0.201; NRI 0.06 and *P*-value = 0.754) (Figure 4).

### Explanatory Power of Δ [LV GLS] in Cardiac Response

**FIGURE 5:**
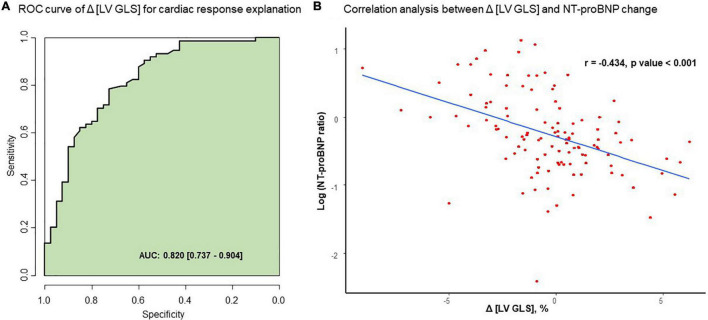
Correlation between Δ [LV GLS] and NT-proBNP change after chemotherapy. **(A)** ROC curve of Δ [LV GLS] for cardiac response explanation. **(B)** Correlation analysis between Δ [LV GLS] and NT-proBNP change. AUC, area under curve; Δ [LV GLS], post-chemotherapy [LV GLS]—pre-chemotherapy [LV GLS]; NT-proBNP ratio, post chemotherapy NT-proBNP/pre chemotherapy NT-proNBP, ROC, receiver operating characteristic.

In the evaluation of cardiac response with ROC analysis, Δ [LV GLS] had good correlation with the presence of cardiac response after chemotherapy (AUC 0.820, 95% CI: 0.737–0.904) (Figure 5A). Baseline [LV GLS] and baseline NT-proBNP level before chemotherapy has significant correlation (*r* = −0.252, *p*-value = 0.006) (Supplementary Figure 1). Δ [LV GLS] and NT-proBNP ratio after chemotherapy also showed modest degree and significant correlation (*r* = −0.434, *p*-value < 0.001) (Figure 5B).

## Discussion

With this study, we investigated longitudinal change in [LV GLS] after the first line of chemotherapy and its association with overall survival and cardiac organ response in cardiac light chain amyloidosis patients. In this study, (1) Δ [LV GLS] had excellent prognostic implications for overall survival in patents with cardiac AL amyloidosis and (2) Δ [LV GLS] after chemotherapy was associated with cardiac organ response.

Among multiple echocardiographic parameters, GLS has been proven to be a strong and independent predictor of prognosis and has additive value to current staging system in patients with cardiac amyloidosis (15, 17, 23). Despite the importance of strain in cardiac amyloidosis, little is known about longitudinal change of [LV GLS] after chemotherapy (24, 25). Since myocardial wall stress caused by amyloid infiltration is reflected by both decreased LV strain (26, 27) and elevated NT-proBNP level, (28) longitudinal change of [LV GLS] after chemotherapy could theoretically be used to predict cardiac response and mortality in patients with cardiac amyloidosis, as verified in the present study.

### Post-chemotherapy Change of [LV GLS] and Echocardiographic Parameters

Diastolic dysfunction by infiltrative protein is the hallmark of cardiac amyloidosis (29, 30), and diastolic dysfunction progressed with disease progression (31). Because progressed cardiac amyloidosis was considered incurable prior to the development of therapeutic agents, there were few papers dealing with improvement in diastolic function after treatment. The Salinaro et al. study (23) addressed longitudinal change of [LV GLS] after chemotherapy. The differences between our study and the Salinaro et al. study was the follow-up of cardiac echo. An additional difference in our study compared to previous studies was that we looked at the cardiac organ response in addition to mortality as an outcome (12, 23). Consistent with previous studies, the diastolic dysfunction parameter, E/e’, and RVSP improved after chemotherapy in the CR group (12, 23). In contrast to the previous study (23), deterioration of [LV GLS] in the non-CR group was evident, which is in line with increase in NT-proBNP level and septal wall thickness in the non-CR group in this study.

### Prognostic Implications of Δ [LV GLS] in Overall Survival and Organ Response

LV wall stress induced by amyloid proteins contributes to both a decrease in [LV GLS] and an increase in NT pro-BNP level in patients with amyloidosis through mechanical infiltration and cytotoxicity. The current staging system includes NT-proBNP and troponin for cardiac involvement, and cardiac response is assessed by changes in NT-proBNP. The correlation between [LV GLS] and NT-proBNP is well proven in patients with myocardial infarction (32) and congestive heart failure (33, 34). This study is meaningful in that it showed that LV GLS and NT-proBNP correlate well in amyloidosis. Incorporating Δ [LV GLS] when assessing cardiac response may be more useful, especially in clinical situation when value of NT pro-BNP is limited, such as acute kidney injury, pulmonary edema, and infection.

Δ [LV GLS] after chemotherapy also has predictive value in overall survival and additive prognostic value to the Mayo 2012 stage + pre-chemotherapy [LV GLS] model. If [LV GLS] is an indicator of cardiac amyloidosis burden before chemotherapy, Δ [LV GLS] is also an indicator of treatment response. Since baseline LV wall stress is already reflected in the NT-proBNP level of the Mayo 2012 stage, pre-chemotherapy [LV GLS] might not have added value to the Mayo 2012 stage model in this study. Also, small number of patients may have resulted in the following results.

With this study, evidence of using Δ [LV GLS] in evaluating cardiac organ response and overall prognosis was prepared for the first time. In addition to NT-proBNP, which is affected by various conditions, if there is no deterioration in the longitudinal change of LV GLS after chemotherapy, it can help in the clinical decision-making process for future treatment.

## Limitation

This study has the limitation of being a single-center retrospective study. First, selection bias could not be avoided, as a feature of retrospective study. Only patients who were able to undergo chemotherapy and those who underwent follow-up echocardiography after chemotherapy were included. Second, although this study was conducted with a small number of patients, all of them had follow-up data on laboratory and echocardiographic markers and outcomes. Compared to previous studies, the number of patients in our cohort is not small. Third, ethnic and regional diversity was limited due to the characteristics of a single-center study. But, since the study was from one of the largest amyloidosis centers in Korea, uniform measurement of laboratory and echocardiographic parameters and standardized chemotherapy were possible. Fourth, the cut-off value of Δ [LV GLS] was obtained using vendor-independent software in this study, but if other types of vendor-dependent software are used, the value could be different. Nevertheless, our result has the important finding that longitudinal change in LV GLS after chemotherapy predicts cardiac response as well as overall survival. Further studies are needed to determine the universal application of this finding.

## Conclusion

In AL amyloidosis patients with cardiac involvement, longitudinal change of LV GLS after chemotherapy showed a significant association with cardiac response and had significant prognostic value.

## Data Availability Statement

The original contributions presented in this study are included in the article/Supplementary Material, further inquiries can be directed to the corresponding author.

## Author Contributions

All authors contributed to the conception and interpretation of data, drafting of the manuscript, revising it critically for important intellectual content, and final approval of the manuscript.

## Conflict of Interest

The authors declare that the research was conducted in the absence of any commercial or financial relationships that could be construed as a potential conflict of interest.

## Publisher’s Note

All claims expressed in this article are solely those of the authors and do not necessarily represent those of their affiliated organizations, or those of the publisher, the editors and the reviewers. Any product that may be evaluated in this article, or claim that may be made by its manufacturer, is not guaranteed or endorsed by the publisher.

## Abbreviations

AL: light chain amyloidosis
CA: cardiac amyloidosis
CI: confidence interval
FLC: free light chain
dFLC: difference between involved and uninvolved free light chains
2D: two-dimensional
IDI: Integrated Discrimination Index
IVST: interventricular septum thickness
GLS: global longitudinal strain
HR: hazard ratio
LV: left ventricular
LVEF: left ventricular ejection fraction
NT-proBNP: N-terminal pro–B-type natriuretic peptide
NYHA: New York Heart Association
NRI: Net Reclassification Improvement
ROC: receiver operating characteristic.
