# Corrigendum: Prognostic implication of longitudinal changes of left ventricular global strain after chemotherapy in cardiac light chain amyloidosis

**DOI:** 10.3389/fcvm.2022.981833

**Published:** 2022-07-20

**Authors:** Minjung Bak, Darae Kim, Jin-Oh Choi, Kihyun Kim, Seok Jin Kim, Eun-Seok Jeon

**Affiliations:** ^1^Division of Cardiology, Department of Medicine, Samsung Medical Center, Heart Vascular Stroke Institute, Sungkyunkwan University School of Medicine, Seoul, South Korea; ^2^Divsion of Hematology and Oncology, Department of Medicine, Samsung Medical Center, Sungkyunkwan University School of Medicine, Seoul, South Korea

**Keywords:** cardiac amyloidosis, global longitudinal strain, cardiac organ response, mortality, chemotherapy

In the published article, there was an error in the Funding statement. [This research was supported by a fund (code: 2019ER690200) by Research of Korea Centers for Disease Control and Prevention]. The correct Funding statement appears below.

## Funding

This research was supported by a fund (code: 2022-ER0507-00) by Research of Korea Disease Control and Prevention Agency.

The authors apologize for this error and state that this does not change the scientific conclusions of the article in any way. The original article has been updated.

## Publisher's note

All claims expressed in this article are solely those of the authors and do not necessarily represent those of their affiliated organizations, or those of the publisher, the editors and the reviewers. Any product that may be evaluated in this article, or claim that may be made by its manufacturer, is not guaranteed or endorsed by the publisher.

