# Supplementary figures and images for: Prognostic Implication of Longitudinal Changes of Left Ventricular Global Strain After Chemotherapy in Cardiac Light Chain Amyloidosis
Source: Front Cardiovasc Med. 2022 Jun 24;9:904878. doi: 10.3389/fcvm.2022.904878 (PMC9263120; doi:10.3389/fcvm.2022.904878)

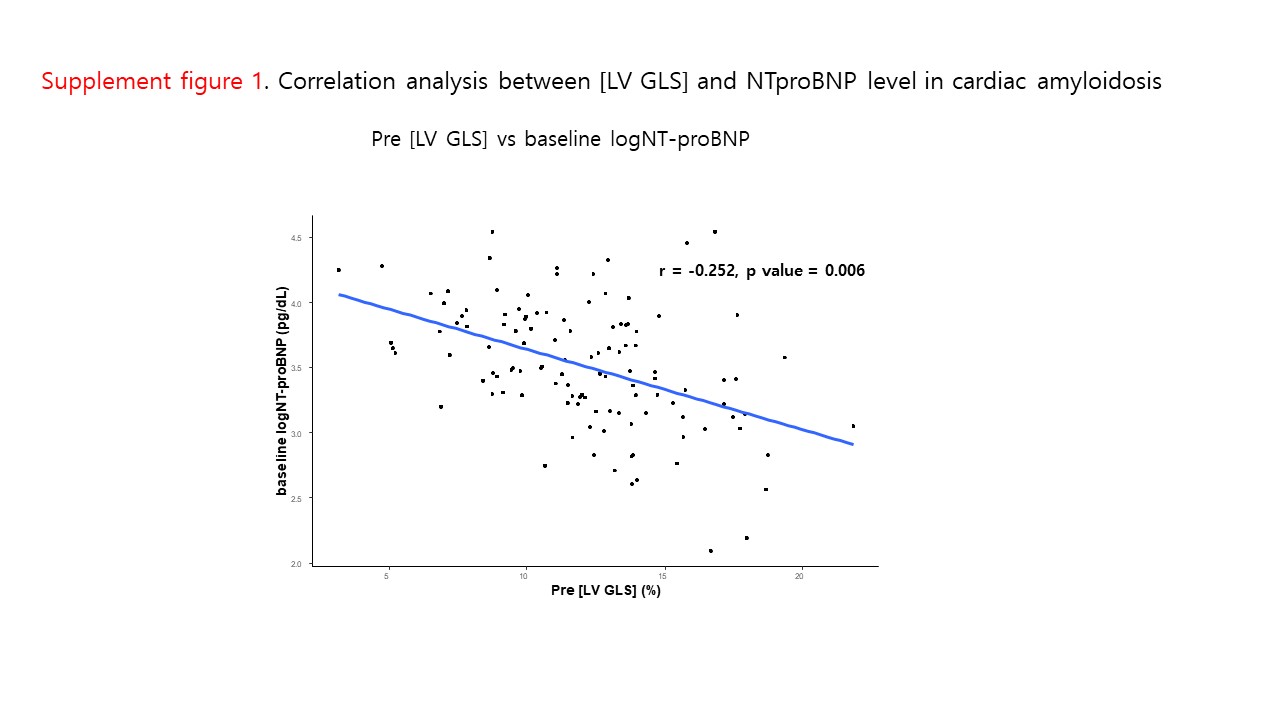

Supplement: Supplementary file 1 [file Image_1.JPEG]
